# The Effect of HIF‐2*α* on the Development of Inflammation

**DOI:** 10.1155/ijin/9092758

**Published:** 2025-12-29

**Authors:** Jiarui Huang, Daohong Zhao

**Affiliations:** ^1^ Department of Orthopaedics, The Second Affiliated Hospital of Kunming Medical University, Kunming, China, kmmc.cn

## Abstract

Hypoxia‐inducible factor‐2*α* (HIF‐2*α*), a key regulator of cellular adaptation to hypoxia, modulates cellular metabolism, allowing cells to survive under hypoxic conditions. In immune responses, infected or inflamed tissues often exhibit hypoxia, and HIF‐2*α* plays a vital role in helping immune cells adapt. HIF‐2*α* also plays a dual and context‐dependent role in inflammation. HIF‐2*α* exhibits both pro‐ and anti‐inflammatory effects in inflammation depending on cell type, disease microenvironment, and signaling pathways. This article describes how HIF‐2*α* regulates immune cell function and its essential role in inflammation, as well as the effects of HIF‐2*α* on the development of inflammation through different signaling pathways. Finally, it explores the potential of HIF‐2*α* as a therapeutic target.

## 1. Introduction

**Figure 1:**
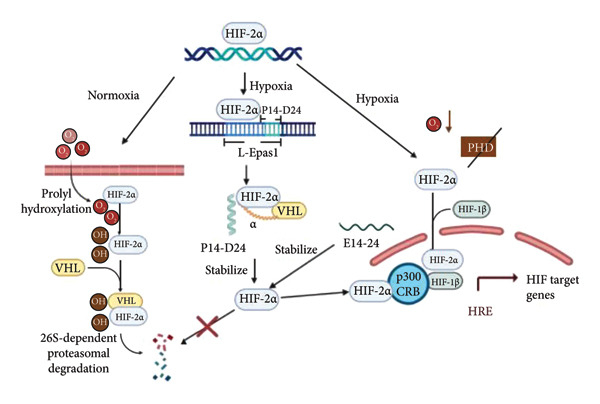
Transcription and activation of HIF‐2*α* occur under hypoxic and normoxic conditions, along with the mechanism by which L‐EPAS1 stabilizes the HIF protein^1^ ^,^ ^2^.

Hypoxia‐inducible factor (HIF) is a heterodimeric transcription factor composed of alpha and beta subunits [1], that is, two helix–loop–helix proteins [2]. Previous studies have identified three related alpha subunit paralogs (HIF‐1*α*, HIF‐2*α*, and HIF‐3*α*) and three isoforms of the beta subunit, also known as the aromatic hydrocarbon receptor nuclear translocator proteins (Arnt1, Arnt2, and Arnt3) [3]. Expression of the alpha subunit is oxygen‐dependent and, therefore, oxygen‐regulated, whereas the beta subunit is constitutively expressed [4]. Three prolyl‐4‐hydroxylase domain enzyme (PHD) subtypes have now been identified in mammals, each of which is encoded by different genes: PHD 1 (also known as egg laying defective gene [EGL] 9 homolog [EGLN] 2 or HIF prolyl hydroxylase [HPH] 3), PHD 2 (EGLN 1 or HPH 2), and PHD 3 (EGLN 3 or HPH 1) [5, 6]. HIF‐2*α* is one of the functional subunits of the HIF family. Under normal oxygen conditions, PHDs in the cytoplasm catalyze the hydroxylation of proline residues in the oxygen‐dependent degradation of the structural domain of HIF‐2*α*, allowing 4‐hydroxyproline to be recognized by the E3 of the von Hippel–Lindau (VHL) protein [7]. By targeting HIF‐2*α* through polyubiquitination by the E3 ubiquitin–ligase complex, ubiquitin ligase enables the proteasome to degrade HIF‐2*α* eventually [8]. Under hypoxic conditions, the activity of PHD and factors that inhibit HIF‐2*α* are inhibited, stabilizing HIF‐2*α* from degradation and enabling it to gradually accumulate in the cytoplasm and translocate to the nucleus, where it dimerizes with the constitutive subunit HIF‐1β/Arnt1 to form a heterodimer. Once formed, the heterodimer will then bind to a specific site of the target gene that is in a region of the target gene’s DNA promoter or enhancer known as the hypoxia response element (HRE) to induce transcription of its target gene, which may include proinflammatory cytokines [9]. Notably, Eric Erquan Zhang found that the presence of a 24‐amino acid insertion sequence (L‐EPAS1) in the EPAS1 (a relatively new name for HIF‐2*α*) protein of the plateau pika also reduces its ubiquitination and enhances the stability of the HIF protein and successfully synthesized a peptide E14‐24 after discovering that the second half of the insertion sequence (P14‐D24) could be conjugated to pVHL through the exploration of molecular mechanisms. A mimetic peptide E14‐24 was successfully synthesized, which was also found to stabilize the HIF‐α protein and induce the expression of the HRE gene [10]. As illustrated in Figure 1, HIF‐2*α* is degraded under normal oxygen conditions (normoxia). This degradation occurs through the oxygen‐dependent hydroxylation of HIF‐2*α* by PHDs, which act on proline residues. Once these prolines are hydroxylated, they are recognized by the VHL E3 ubiquitin ligase, leading to ubiquitination and rapid degradation by the proteasome. In contrast, under low oxygen conditions (hypoxia), the activity of PHDs is inhibited due to the absence of oxygen, their necessary co‐substrate. As a result, HIF‐2*α* can accumulate and move into the nucleus; the bHLH domain of HIF‐2*α* binds to HREs, while the CTAD recruits co‐regulators CRB/P300. The 24‐amino acid insertion sequence (L‐EPAS1) in the EPAS1 protein reduces ubiquitination, enhancing HIF stability. The latter half (P14‐D24) can conjugate with pVHL, leading to the synthesis of the peptide E14‐24. This mimetic peptide stabilizes the HIF‐2*α* protein and activates the expression of the important HRE gene.

## 2. Biological Functions of HIF‐2*α*

HIF‐2*α* is a key mediator of cellular adaptation to hypoxia and plays a vital role in regulating cellular metabolism. It has been demonstrated that HIF‐2*α* mediates the adaptive response to hypoxia in tumor cells. A well‐known example of this is found by Swethajit et al. [11], who concluded that HIF‐2*α* enhances c‐Myc transcriptional activity (HIF‐2+) and promotes tumor growth by maintaining low levels of glycolysis, allowing for more mitochondrial metabolism and tolerance to ROS‐induced DNA damage for a more oxidative phenotype. Several recent studies have revealed that HIF‐2*α* regulates hepatic erythropoietin (EPO) production and lipid metabolism. HIF‐2*α* is required for physiological EPO expression and erythropoiesis in infant livers [12]. In contrast to infants, increased HIF‐2*α* activity will lead to erythrocytosis and vascular tumorigenesis in adults [13, 14]. Notably, activation of HIF‐2*α* will lead to impaired fatty acid *β*‐oxidation, decreased lipogenic gene expression, and increased lipid storage capacity, ultimately progressing to severe hepatic steatosis [15]. Interestingly, HIF‐2*α* plays a crucial role in iron absorption, promoting iron absorption by regulating the expression of divalent metal ion transport protein 1 (DMT1), duodenal cytochrome b561 (DCYTB), and iron transport protein (FPN) [16]. In conclusion, HIF‐2*α* is essential for various physiological processes involved in regulating cellular metabolism, angiogenesis, glycolysis, and iron metabolism [17–20].

**Figure 2:**
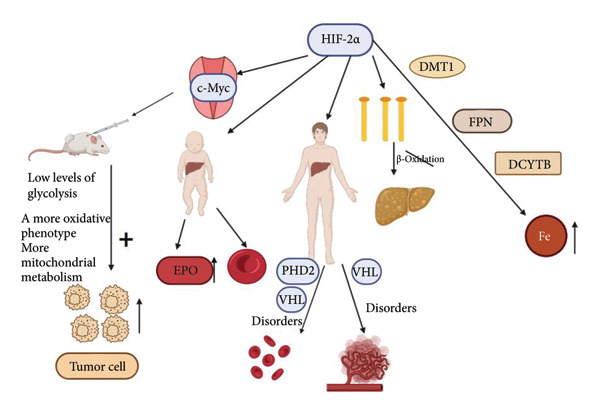
Biological functions of HIF‐2*α* in the liver and various cells^3^ ^,^ ^4^.

As illustrated in Figure 2, HIF‐2*α* enhances the transcriptional activity of c‐Myc (HIF‐2+) and promotes tumor growth by maintaining low levels of glycolysis. This allows for increased mitochondrial metabolism and improved tolerance to ROS‐induced DNA damage, contributing to a more oxidative phenotype. In infants, HIF‐2*α* is essential for the expression of EPO and the process of erythropoiesis in the liver. In adults, disturbances in blood homeostasis caused by the dysregulation of prolyl hydroxylase domain protein 2 (PHD2) and VHL can lead to erythrocytosis and vascular tumors. Additionally, the activation of HIF‐2*α* can impair fatty acid *β*‐oxidation, decrease lipogenic gene expression, and increase lipid storage capacity, ultimately leading to severe hepatic steatosis. Moreover, HIF‐2*α* plays a role in promoting iron absorption by regulating the expression of DMT1, DCYTB, and FPN.

## 3. The Relationship Between HIF‐2*α* and Inflammation

Inflammation is a process of immune response due to microbial invasion (especially bacterial infections), in which the invading pathogen first triggers receptors of the innate immune system such as Toll‐like receptor (TLR) and NOD (nucleotide‐binding oligomerization structural domain protein)‐like receptor (NLR). These receptors mediate the onset of infection recognition by macrophages and mast cells, leading to chemokines such as macrophage inflammatory factors (MIP‐1a and MIP‐1β), interleukin‐8 (IL‐8), inflammatory cytokines such as interleukin‐1β (IL‐1β), tumor necrosis factor‐α (TNF‐α), interleukin‐6 (IL‐6), and vasoactive amines, among many other inflammatory mediators, which will give rise to localized inflammatory exudates: Neutrophils confined to blood vessels pass through the small postcapillary veins into the extravascular tissue at the site of infection (or injury). The activated vascular endothelium allows selective extravasation of neutrophils, which are activated upon arrival at the site of the affected tissue and then form substances such as ROS and reactive nitrogen species, which are effectors that will indiscriminately damage pathogens and the human body, resulting in damage to human tissues. While the sites of pathogen‐ and microbe‐induced tissue inflammatory responses are usually in a hypoxic environment, this is due to the significant changes in metabolic activity, energy supply, and demand at the site of inflammation, resulting in reduced oxygen delivery and/or availability, leading to inflammation‐associated tissue hypoxia and metabolic acidosis. To date, the role of HIF‐1*α* in various diseases has been extensively investigated, and with the role of HIF‐1*α* in inflammation having been gradually studied and understood, researchers have become more interested in the study of HIF‐2*α*. HIF‐2*α* is believed to be essential for regulating cytokine expression, macrophage migration, and response to inflammatory stimuli.

## 4. Inflammatory Diseases in Which HIF‐2*α* Exerts an Anti‐Inflammatory Effect

In recent years, researchers have found that HIF‐2*α* plays a role in inhibiting proinflammatory responses in many inflammatory reactions. In the study of lung ischemia‐reperfusion injury (LIRI), Ge et al. concluded that Aquaporin‐1 (AQP1) plays a vital role in the long‐term repair of lung ischemia‐reperfusion (IR) injury and that it promotes angiogenesis of pulmonary microvascular endothelial cells (PMVECs) and the repair of lung injury, mainly by stabilizing the expression level of HIF‐2*α* [21]. miRNAs are essential regulators of gene expression and play a key role in the pathogenesis of lung IR injury and organ exclusion pathogenesis [22]. miR‐223 is highly expressed in lung IR injury. It exacerbates the lung IR injury by targeting and inhibiting the expression of HIF2*α* and *β*‐linker proteins in PMVECs, which promote autophagy, suggesting that HIF‐2*α* protects PMVECs [22]. In acute respiratory distress syndrome (ARDS), HIF‐2*α* is induced through the induction of expression of vascular endothelial protein tyrosine phosphatase (VE‐PTP) and enhances the integrity of adherens junctions (AJs) between endothelial cells, thereby improving vascular endothelial barrier function and preventing edema formation and inflammatory cell infiltration [23]. Proper [24] led a team of researchers to study hard metal lung disease (HMLD) due to cobalt exposure by specifically knockdown of HIF‐2*α* in alveolar epithelial cells, which was found to exacerbate inflammatory cell infiltration, confirming that HIF‐2*α* plays an important role in lung repair mechanisms. Gastrointestinal mucosal hypoxia is a common feature of the mucosa at the site of inflammation in inflammatory bowel disease (IBD) as a result of a combination of increased oxygen consumption by immune cells (e.g., monocytes, macrophages, dendritic cells, and neutrophils) recruited after activation of inflammatory responses and resident cells in the inflamed mucosa, as well as decreased tissue perfusion due to microthrombus formation at the site of chronic inflammation [25, 26]. Kerber et al. examined the expression levels of inflammatory factors in HIF‐1*α*, HIF‐2*α*, and HIF‐1*α*/HIF‐2α‐double knockout acute colitis (DSS) mice by conditionally knocking out HIF‐1*α* and HIF‐2*α* in myeloid cells, respectively. They showed that conditional knockout of HIF‐2*α* exacerbated neutrophil recruitment and induced the expression of proinflammatory cytokines [27]. However, the opposite results were observed in intestinal cancers, which are different from IBD in a number of respects. Colon cancer, which is strongly linked to IBD‐associated chronic inflammation, is a significant risk factor for colon cancer, and HIF‐2*α* deletion reduced the number of colon tumors and tumor load and increased apoptosis of tumor cells. Conversely, overexpression of HIF‐2*α* increased colon tumorigenesis and tumor cell proliferation. This opposite result of HIF‐2*α* regulation of inflammation and cancer in the intestine is due to the fact that in the colon tumor microenvironment, HIF‐2*α* is able to directly regulate a potent neutrophil chemokine, CXCL1, resulting in overexpression of HIF‐2*α* promoting the recruitment of neutrophils to colon tumors and promoting tumor proliferation [28]. In conclusion, in the intestinal inflammatory microenvironment, HIF‐2*α* plays a vital role in initiating inflammation and the abatement of injury, whereas in the intestinal tumor microenvironment, HIF‐2*α* is critical for tumorigenesis and development.

## 5. Inflammatory Diseases in Which HIF‐2*α* Exerts a Proinflammatory Effect

Hypoxia can also occur in the adipose tissue of obese individuals, and chronic inflammation is a potential etiology of obesity‐associated heart disease, although the mechanisms remain to be elucidated [29]. Lin et al. found that the HIF pathway can be activated by explicitly knocking down the VHL gene in mouse adipocytes, leading to accumulation of HIF‐1*α* and HIF‐2*α* proteins in adipocytes, thereby mimicking hypoxic signaling [30]. These investigators propose that the link between obesity and cardiomyopathy may be through changes in the microenvironment of adipose tissue (e.g., hypoxia) leading to HIF‐2*α* induction, which in turn triggers inflammation and cardiomyopathy, and that inflammation in adipose tissue is a significant cause of pathologic cardiac hypertrophy and heart failure, with HIF‐2*α* activation being a key mechanism in this process [31]. Articular cartilage is subjected to a hypoxic environment throughout, and HIF‐2*α* is mainly expressed in highly differentiated chondrocytes, with significantly elevated levels in human and mouse osteoarthritis (OA) chondrocytes when OA occurs [32]. Hiroaki’s study demonstrated that at a hydrostatic pressure of 5 MPa, expression of HIF‐2*α* could be upregulated independent of NF‐κB, thereby inducing matrix metalloproteinases (MMPs), which causes the expression of MMP‐13 and MMP‐3, leading to cartilage degeneration and exacerbation of OA [33]. Gastroesophageal reflux esophagitis (GERD) and periodontitis are mostly nicotine‐mediated inflammatory injuries. Their studies have shown that HIF‐2*α* promotes the production of proinflammatory cytokines in esophageal epithelial cells and human periodontal ligament cells (PDLCs), which exacerbate the symptoms of GERD or periodontitis [34]. Polymorphonuclear neutrophils (PMNs) are a key cell type in tissue damage in acute and chronic inflammatory diseases [35]. Thompson concluded that HIF‐2*α* plays a role in prolonging the survival and inflammatory response in neutrophils by examining the role of HIF‐2*α* in PMN function in humans, mice, and zebrafish and suggested the possibility of treating chronic inflammatory diseases by modulating HIF‐2*α* [36].

Overall, these studies support the view that the role of HIF‐2*α* in inflammatory diseases remains indecisive. In endothelial cells and alveolar epithelial cells, HIF‐2*α* plays a protective and reparative role, while in different types of cellular inflammation, HIF‐2*α* promotes the development of inflammation. The mechanisms involved need to be further explored and elucidated.

## 6. Role of HIF‐2*α* in Inflammatory Diseases by Regulating Macrophage Activity

**Figure 3:**
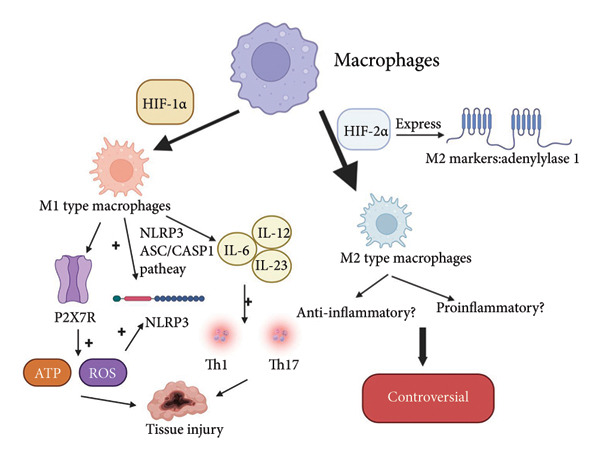
HIF‐1*α* promotes the polarization of macrophages toward the M1 type, while HIF‐2*α* promotes their polarization toward the M2 type. M1‐type macrophages worsen the inflammatory response and cause tissue damage, while the role of M2‐type macrophages remains controversial^5^ ^,^ ^6^.

Macrophages are formed by the differentiation of bone marrow cells, which are called monocytes that circulate in the bloodstream before eventually differentiating into “resident” tissue macrophages that migrate to infected tissues [37]. They play a vital role in responding to bacterial, viral, and other pathogen infections by migrating to damaged tissues to exert anti‐infective effects [38]. Interestingly, these cells accumulate in large numbers within areas of hypoxia in various inflammatory diseases such as bacterial infections and wounds, suggesting that hypoxia regulates macrophage bioactivity in inflammatory environments [39]. HIF‐2*α* is important for macrophage migration. For example, HIF‐2*α* is expressed in tumor‐associated macrophages (TAMs), and the absence of HIF‐2*α* in TAMs results in decreased expression of chemotactic receptors and reduced migration and infiltration of TAMs [39]. Macrophages exhibit different functions according to various stimuli imposed by the local environment. HIF‐1*α* and HIF‐2*α* play distinct roles in macrophage polarization. Overexpression of HIF‐1*α* in macrophages promotes the expression of genes related to the M1 type (classically activated). In contrast, the related transcription factor HIF‐2*α* promotes M2 polarization through the expression of M2 (alternatively activated) markers, such as adenylylcyclase 1 (also known as adenylylase 1), but HIF‐1*α* and HIF‐2*α* are not required for macrophage polarization in aseptic inflammation models [38, 40]. Hypoxia promotes the generation of M1‐type macrophages, M1‐type macrophages acting on ATP and ROS produced by the P2X7R ion channel receptor or through the NLRP3/ASC/CASP1 pathway can trigger activation of the NLRP3 inflammasome, and M1 macrophages derived proinflammatory cytokines (i.e., IL‐6, IL‐12, and IL‐23) exacerbated tissue injury by enhancing the Th1 and Th17 immune responses [41], whereas M2 macrophages suppressed the inflammatory response and promoted the repair of the injury site [38]. Moreover, it has been demonstrated in studies of two HIF‐α isoform double knockout mouse models of acute colitis that HIF‐1*α* and HIF‐2*α* in macrophages play opposite roles in acute colitis, suggesting that the relevant expression of HIF‐2*α* should have an anti‐inflammatory effect [27]. However, the function of the M2 type of macrophage is still controversial. A study of the HIF‐2*α* gene knockout mouse model by Imtiyaz et al. concluded that under hypoxia, HIF‐2*α* acts as a key transcriptional regulator in macrophages to amplify the expression of inflammatory cytokines and other inflammatory responses such as leukocyte infiltration [42]. Still, Li et al. found that stabilizing macrophage HIF‐2*α* expression significantly attenuated inflammatory vesicle activation, and the team concluded that the inconsistent results were due to the objective conditions, such as the mice and experimental conditions [43]. In subsequent studies by different scholars after Imtiyaz, Jeekani et al. studied nonalcoholic steatohepatitis (NASH), a chronic liver disease. They concluded that macrophage isoforms of HIF‐2*α* promoted proinflammatory activation of recruited hepatic macrophages (RHM), leading to the development of NASH [44]. An anti‐inflammatory role of macrophage HIF‐2*α* has also been argued in studies of other different inflammatory responses, confirming that the role of macrophage HIF‐2*α* can be to inhibit the proinflammatory response [45]. The role and mechanism of HIF‐2*α* in activating inflammatory vesicles have still not been fully elucidated, and therefore, further studies are needed to validate the role of macrophage HIF‐2*α*. Figure 3 illustrates how HIF‐2*α* promotes the polarization of macrophages into the M2 type. As illustrated in the figure, the overexpression of HIF‐1*α* in macrophages enhances the expression of M1‐type (classically activated) related genes. In contrast, the transcription factor HIF‐2*α* facilitates M2 polarization by inducing the expression of M2 (alternatively activated) markers, such as adenylate cyclase 1. M1‐type macrophages respond to ATP and reactive oxygen species (ROS) generated by the P2X7R ion channel receptor or via the NLRP3/ASC/CASP1 pathway. The activation of NLRP3 inflammasomes can be triggered in this context, leading to the release of proinflammatory cytokines by M1 macrophages, including IL‐6, IL‐12, and IL‐23. These cytokines exacerbate tissue damage by amplifying Th1 and Th17 immune responses. The function of M2‐type macrophages remains controversial, and the role and mechanisms of HIF‐2*α* in the activation of inflammasomes are still not fully understood.

## 7. Role of HIF‐2*α* in the Regulation of Inflammation by the NF‐κB Pathway

Nuclear factor‐κB (NF‐κB), a collective term for a family of transcription factors capable of being induced, was first discovered and proposed by Ranjan Sen and David Baltimore in 1986. It is a nuclear factor that regulates a large number of genes involved in the immune and inflammatory response process [46]. The activation of NF‐κB occurs mainly through two different signaling pathways, the classical and the nonclassical (or alternative) pathways. The classical pathway is activated through the IKKα/β/*γ* complex [47], whereas the nonclassical pathway involves NIK‐mediated activation of IKKα homodimers [48, 49]. Activation of the NF‐κB pathway is widely recognized as a feature of inflammation, and its function in modulating the inflammatory response is well‐established. NF‐κB not only mediates the induction of a wide range of proinflammatory genes in innate cells and regulates the activation, differentiation, and effector functions of inflammatory T cells but also regulates the activation of inflammatory vesicles [50]. Dysregulation of NF‐κB activation is a hallmark of chronic inflammatory diseases [51]. In the inflammatory microenvironment, HIF and NF‐κB undergo extensive crosstalk, and the crosstalk is mutual, with NF‐κB inducing HIF and, in turn, HIF regulating NF‐κB, especially in the inflammatory microenvironment [52]. The NF‐κB family member RELA is a known potent inducer of HIF‐2*α* expression [53]. Previous studies have indicated that hypoxia activates HIF and NF‐κB gene expression primarily through the classical activation pathway of the NF‐κB signaling pathway [47]. The inflammation‐related expression of HIF‐2*α* in the heart, lungs, and kidneys has been extensively studied. Kobayashi et al. observed the modulatory role of medullary cell‐derived HIF in unilateral ureteral obstruction (UUO)‐induced renal injury through global activation of HIF, and these authors concluded that HIF modulates inflammatory responses in UUO. The regulatory role of HIF in UUO‐induced renal injury was investigated by these authors, who concluded that medullary cells inhibited PHD 1, and PHD 2 inhibited IκB kinase‐β (IKK‐β) under hypoxic conditions, which led to the activation of NF‐κB, which stimulated the transcription of HIF, whose anti‐inflammatory effects were activated and reduced UUO‐induced renal inflammation [54]. Subsequently, Kapitsinou et al. further investigated the functions of HIF‐1*α* and HIF‐2*α* in renal endothelial cells of mice with renal ischemia/reperfusion injury (IRI) in renal injury disease. They concluded that endothelial cells have a key role in renoprotection, in which PHD exerts a nondependent function of HIF to regulate NF‐κB negatively, leading to the activation of NF‐κB signaling and HIF prolyl hydroxylase inhibition, leading to modulation of apoptotic responses to suppress inflammation and promote tissue repair to protect against IRI‐ and UUO‐induced kidney injury [55]. He et al. further explored the mechanism of HIF‐2*α* in endothelial cells to protect against IRI. They discovered that the accumulation of HIF‐2*α* in the endothelial cells was increased in the endothelial cells, that the HIF‐2*α* accumulation in the endothelial cells was increased in the endothelial cells, and that HIF‐2*α* accumulation in the endothelial cells in the endothelial cells was notably increased. In their study of the mechanism by which lipopolysaccharide (LPS) pretreatment can attenuate renal IRI, they found that HIF‐2*α* accumulation in endothelial cells further confirmed that LPS relies on the nuclear translocation of NF‐κB to upregulate the transcriptional level of HIF‐2*α* and regulate the expression of nitric oxide synthase (NOS), which in turn increases the production of nitric oxide (NO), thus improving the renal microcirculation and attenuating IRI [56, 57]. In conclusion, both HIF and NF‐κB are capable of inducing the expression of pro‐inflammatory cytokines. The elevated levels of HIF‐2*α* in endothelial cells rely on the induction of NF‐κB, and ultimately, HIF‐2*α* exerts anti‐inflammatory effects and promotes tissue damage repair. As mentioned earlier, the anti‐inflammatory effects of HIF‐2*α* also hold in lung endothelial cell lesion diseases [21, 23, 24]. The promotion of NASH by HIF‐2*α* occurs not only through the pro‐inflammatory activation of hepatic macrophages but also through the advancement of NASH to hepatic fibrosis [44]. This progression is mediated by the NF‐κB signaling pathway, which upregulates the expression of inflammation‐related genes. These findings were confirmed in studies conducted by Cai et al. after establishing a NASH mouse model [58]. As mentioned previously, in studies of obesity‐related cardiomyopathy, activation of HIF‐2*α* in adipocytes leads to increased expression of inflammatory factors and chemokines that promote cardiac hypertrophy through activation of the NF‐κB and NFAT pathways in the heart [30]. In a study examining OA of the temporomandibular joint, researchers found that mechanical stress significantly increased the expression of NF‐κB, HIF‐2*α*, and related downstream factors. To further investigate this, they employed RNA interference targeting NF‐κB to confirm that the upstream NF‐κB pathway plays a regulatory role in HIF‐2*α*. This regulation contributes to cartilage degradation and worsens inflammation [59]. Interestingly, the inflammatory response involving HIF‐2*α*, which is mainly mediated through the NF‐κB pathway, plays different roles. This suggests that the proinflammatory and anti‐inflammatory effects of HIF‐2*α* in inflammation are influenced by factors beyond just the signaling pathways.

**Figure 4:**
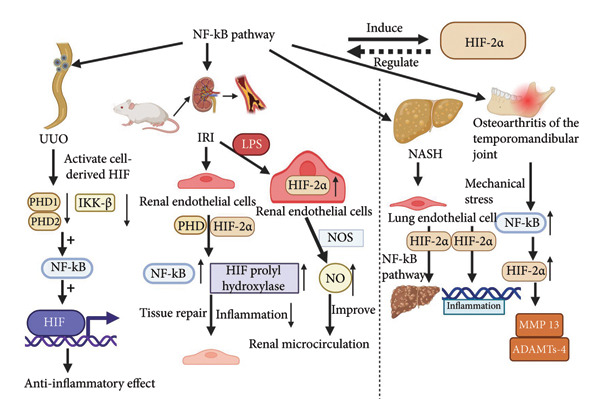
The proinflammatory or anti‐inflammatory effects of HIF‐2*α* on the mutual induction and regulation of the NF‐κB pathway in various diseases^7^ ^,^ ^8^.

Figure 4 illustrates the proinflammatory and anti‐inflammatory effects of HIF‐2*α* on the mutual induction and regulation of the NF‐κB pathway across various diseases. As illustrated in the figure, in cases of UUO that lead to kidney injury, myeloid cells suppress the activity of PHD 1 and 2 under hypoxic conditions. This suppression inhibits IKK‐β, resulting in the activation of NF‐κB. NF‐κB then stimulates the transcription of HIF, which has anti‐inflammatory effects and reduces renal inflammation caused by UUO. Additionally, in the renal endothelial cells of mice subjected to renal IRI, HIF‐2*α* plays a critical role in nephroprotection. Hepatocyte growth factor (HD) exerts a function independent of HIF, serving to negatively regulate NF‐κB. This regulation leads to the activation of NF‐κB signaling and affects apoptotic responses by inhibiting prolyl hydroxylase activity. Consequently, this inhibition reduces inflammation and promotes tissue repair, thereby offering protection against renal injury induced by IRI and UUO. The mechanism by which LPS pretreatment can reduce renal IRI involves the accumulation of HIF‐2*α* in endothelial cells. This process depends on the nuclear translocation of NF‐κB to enhance the transcriptional levels of HIF‐2*α*, which in turn regulates NOS expression and increases NO production. As a result, renal microcirculation improves, mitigating the effects of IRI. Additionally, the promoting effect of HIF‐2*α* on NASH may be mediated by the NF‐κB signaling pathway, which facilitates the progression of NASH to liver fibrosis and increases the expression of inflammation‐related genes. In the case of OA of the temporomandibular joint, there is a significant increase in the expression of NF‐κB, HIF‐2*α*, and downstream degradation factors such as MMP‐13 and ADAMTS‐4. These factors are induced by mechanical stress, leading to cartilage degradation and exacerbating inflammation.

## 8. Role of HIF‐2*α* in the Regulation of Inflammation by Other Signaling Pathways

In a study of acute liver injury (AILI) caused by acetaminophen (APAP) overdose, Gao et al. found that HIF‐2*α* was stabilized in APAP‐treated hepatic macrophages, which subsequently reprogrammed the macrophages to produce the key downstream target gene IL‐6, which protects the liver from injury through activation of the IL‐6‐Janus kinase–signal transducer and activator of transcription (JAK–STAT) signaling pathway in the hepatocytes [60]. In myocardial ischemia‐reperfusion injury (MIRI), HIF‐2*α* regulates IL‐6 in cardiomyocytes by transcriptionally activating it. This activation triggers the PI3K/Akt and STAT3 signaling pathways, reducing infarct size during myocardial ischemia/reperfusion (MI/R) and providing protection to the myocardium. Bae et al. studied the effects of inflammatory cytokines, extracellular matrix (ECM)‐destroying enzymes, and osteoblast differentiation in nicotine‐ and LPS‐stimulated human PDLCs and proposed that HIF‐2a inhibitors and HIF‐2a silencing treatments blocked nicotine‐ and LPS‐induced activation of Akt, JAK 2, and STAT 3 extracellular signaling‐regulated kinases (ERKs) and c‐Jun NH 2‐terminal kinases (JNKs) phosphorylation, but not p38 isoforms (p38s), as well as activation of NF‐κB and AP‐1 components, suggesting that HIF‐2*α* acts as a proinflammatory agent in periodontal disease, with a pathway of action that includes, in addition to activation of NF‐κB, MAPK, phosphatidylinositol‐3 protein kinase (PI3K) pathway, and JAK–STAT pathway [61]. Currently, the relationship between these pathways and HIF‐2*α* has been studied more in terms of tumor proliferation and regulation of cell growth and development, and more research is needed to elucidate the mechanism of action in inflammatory diseases.

**Figure 5:**
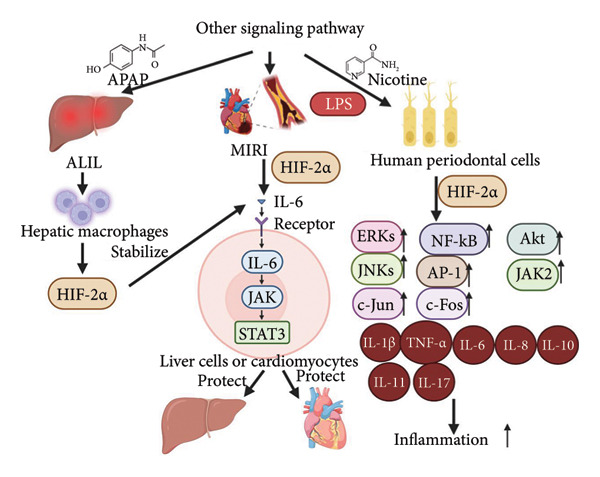
The role of HIF‐2*α* in regulating inflammation by modulating signaling pathways beyond macrophage activation and the NF‐κB pathway^9^ ^,^ ^10^.

Figure 5 demonstrates how HIF‐2*α* regulates inflammation by influencing signaling pathways beyond macrophage activation and the NF‐κB pathway. As illustrated in the figure, in cases of AILI caused by an overdose of APAP, HIF‐2*α* plays a crucial role in reprogramming macrophages to produce the important downstream target gene IL‐6. This production helps protect the liver by activating the IL‐6‐JAK–STAT3 signaling pathway in hepatocytes. Similarly, in MIRI, HIF‐2*α* transcriptionally regulates IL‐6 in cardiomyocytes, which activates the PI3K/Akt and STAT3 signaling pathways, thereby protecting the myocardium. HIF‐2*α* levels increase in human PDLCs when stimulated by nicotine and LPS, in a manner that is both dose‐ and time‐dependent. Additionally, HIF‐2*α* is involved in various signaling pathways, including MAPK/ERK, NF‐κB, and JAK/STAT3. This involvement occurs through the activation of several proteins, such as Akt, JAK2, and STAT3, as well as ERK, JNK, MAPK, nuclear factor‐κB, c‐Jun, c‐Fos, and proinflammatory cytokines.

## 9. Factors Affecting the Anti‐Inflammatory or Proinflammatory Effects of HIF‐2*α*

Summarizing the studies of the above scholars, HIF‐2*α* cannot be categorized as a proinflammatory or anti‐inflammatory factor, and its proinflammatory and anti‐inflammatory effects in inflammation depend not only on the type of inflammation (acute or chronic) but also on the cell type, the inflammatory microenvironment, the downstream signaling pathway, and the disease background. As mentioned previously, in APAP‐induced AILI, HIF‐2*α* exhibited anti‐inflammatory effects in hepatic macrophages by inducing IL‐6 production and activating the STAT3 signaling pathway to protect the liver from acute injury [60]. In contrast, in the chronic inflammatory disease NASH, HIF‐2*α* promotes the replacement of embryonic hepatic koilocytes by bone marrow‐derived RHMs, which are high in proinflammatory genes, by activating the PI3K/Akt/mTOR signaling pathway leading to decreased expression of lysosomal and phagocytic genes, and HIF‐2*α* can also activate the NF‐κB in human hepatocellular carcinoma cells (HepG2) signaling pathway to promote liver fibrosis and inflammation [44, 58]. In nicotine‐induced GERD, HIF‐2*α* in esophageal epithelial cells activated the NF‐κB signaling pathway and stimulated the production of proinflammatory cytokines [34]; in hypoxia‐induced OA, HIF‐2*α* mediated primary cilia loss in primary chondrocytes of mice under hypoxic conditions via the HIF‐2*α*/AURKA/NEDD9 pathway, which facilitated the development of OA [62]. Therefore, there is no critical and well‐recognized determinant for the role of HIF‐2*α*, and relatively more crucial may be the cell type and the specific molecular pathways it regulates. This is evident in the case of PMVECs and alveolar epithelium, where HIF‐2*α* plays a protective and reparative role against injury caused by inflammatory lung diseases [21, 23, 24], as well as for the role of renal endothelial cell HIF‐2*α* [54–57]. These findings cannot be extrapolated to all patients. Therefore, these results need to be interpreted with caution. More studies are necessary to continuously investigate and explore the exact factors that influence the anti‐inflammatory or proinflammatory effects of HIF‐2*α*.

## 10. HIF‐2*α* Is Involved in Promoting the Progression of Other Diseases

The inflammatory microenvironment is characterized by hypoxia, a condition of reduced oxygen availability. Cancer cells can thrive under these hypoxic conditions. In the tumor microenvironment, both hypoxia and macrophage accumulation are characteristic features [63, 64]. HIF‐2*α* has been shown to promote cancer cell growth and angiogenesis across various cancer types. By studying TAMs in mice, some researchers have concluded that hypoxia‐induced macrophages protect HIF‐2*α* from proteasomal degradation and enhance VEGF mRNA expression via the PI3K/Akt signaling pathway, thereby promoting tumor growth, metastasis, and angiogenesis [65]. After hypoxic intervention in human neuroblastoma cell lines (SK‐N‐BE(2)c, SH‐SY5Y, SH‐EP, IMR‐32, KCN‐69n), the researchers found that elevated HIF‐2*α* transcript levels were independent of Akt and mTORC1 signaling and dependent on PI3K/mTORC2 signaling. At the same time, the protein and mRNA levels of VEGF‐A, a target gene of HIF‐2*α*, were also elevated, thus accelerating tumor cell growth [66]. In studies of HepG2, the transcriptional level of HIF‐2*α* was elevated after phosphorylation modification by casein kinase 1*δ* (CK1*δ*; also known as CSNK1D), which enhances the secretion of EPO from HepG2 under hypoxia, and EPO acts on the EPO receptor in the vascular endothelium to promote angiogenesis and HepG2 growth [67]. In addition, exogenous acetate promoted HIF‐2*α* acetylation in an acetate‐dependent acetyl coenzyme A synthase 2 (Acss2) manner, induced the expression of the EPO gene in HepG2, and stimulated erythropoiesis [68]. Acss2/HIF‐2*α* signaling was abnormally active in hypoxia and glucose deficiency and accelerated cell proliferation and colony formation in fibrosarcoma cells and colon cancer cells, migration and invasion in fibrosarcoma and colon cancer cells, and Acss2/HIF‐2α‐dependent cancer cells grown in mice showed faster tumor growth [69, 70]. Clear cell renal cell carcinoma (ccRCC) is a highly vascularized tumor, and sunitinib, a widely accepted first‐line therapeutic agent, inhibits neovascularization by blocking VEGFRs in ccRCC [71]. As more patients develop drug resistance in the clinic, researchers have found that HIF‐2*α* transcriptionally targets the hypoxia‐responsive element of the Polo‐like kinase 1 (Plk1) promoter, leading to high Plk1 expression and conferring a resistant phenotype to targeted therapeutic agents such as sunitinib [72]. In conclusion, HIF‐2*α*, as one of the most critical stress signals in the tumor microenvironment under hypoxia, plays a role in promoting further disease progression and deterioration in many cancer diseases.

**Figure 6:**
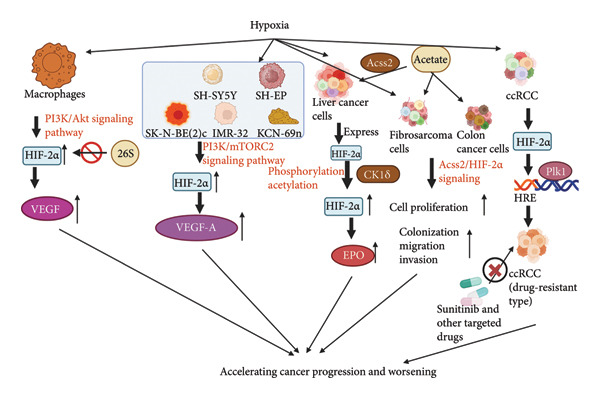
The mechanisms and signaling pathways through which HIF‐2*α* promotes the progression of various types of cancer^11^ ^,^ ^12^.

Figure 6 summarizes the mechanisms and signaling pathways through which HIF‐2*α* promotes the progression of various cancer types. Hypoxia‐induced TAMs stabilize HIF‐2*α* protein via the PI3K/Akt signaling pathway, thereby enhancing VEGF expression. In hypoxia‐treated human neuroblastoma cell lines, the transcription of HIF‐2*α* and its downstream target gene VEGF‐A depends on the PI3K/mTORC2 signaling pathway. In HepG2, the transcription of HIF‐2*α* increases following its phosphorylation by CK1*δ*. Additionally, exogenous acetate promotes the acetylation of HIF‐2*α* in an Acss2‐dependent manner. Both of these pathways enhance the secretion of EPO in hypoxic conditions typical of HepG2. Moreover, HIF‐2*α* transcription targets the hypoxia‐responsive element within the Plk1 promoter in ccRCC, which leads to elevated expression of Plk1. This increased expression contributes to a drug‐resistant phenotype in ccRCC, complicating treatment outcomes with targeted therapies.

## 11. HIF‐2*α* as a Therapeutic Target in Inflammatory Diseases

The traditional treatment strategy for various inflammatory diseases has been to use high doses of hormones, immunosuppressants, or biologics to inhibit inflammation and slow its progression [73–75]. Thankfully, these drugs have worked well for most inflammatory diseases. However, regardless of which drug is used, there are many shortcomings, such as the tendency to relapse after the drug is reduced, the patient’s dependence on hormonal drugs, resistance or intolerance to a particular drug, or the high price of the drug that the patient cannot afford, as well as a host of other clinical problems and complications, such as serious opportunistic infections or the weakening or deterioration of the patient’s original organs or tissues, which prevents patients from regaining their previous standard of living before the onset of the inflammatory disease [76, 77]. Therefore, if drugs that target and inhibit certain inflammatory diseases or inflammatory factors can be developed, the occurrence of side effects and complications after the use of drugs can be reduced. HIF‐2*α* has an essential impact on developing many types of inflammatory diseases. Asthma is characterized by airway inflammation and allergic reactions. Sook Young Lee and Su‐Mi Chung administered Neovastat (AE‐941) after establishing a mouse model of asthma, and they assessed a decrease in the expression of VEGF and HIF‐2*α* in lung tissues, concluding that Neovastat significantly attenuated the airway inflammation in asthmatic mice by inhibiting the expression of VEGF and HIF‐2*α*. Airway inflammation in asthmatic mice and the inhibition of HIF‐2*α* may be one of the key mechanisms underlying the anti‐inflammatory effects of Neovastat [78]. Another example of targeted inhibition of HIF‐2*α* expression is that Kong et al. established a mouse model of experimental colitis and treated mice with Roxadustat (FG‐4592). They found that Roxadustat ameliorated the symptoms of mice by increasing the level of HIF‐2*α*, decreasing the level of inflammatory cytokines, inhibiting the polarization of M1, and increasing the polarization of M2 macrophages in the colonic tissues, which is expected to offer an alternative therapeutic option [79]. Hydrogels are highly regarded as effective biomaterials, providing excellent biocompatibility and efficient drug delivery [80]. In the study of intervertebral disc degeneration (IVDD), researchers developed a composite gel called CAP‐sEXOs@Gel to inhibit inflammation in cartilage endplate cells (CEPC) and nucleus pulposus cells (NPC). This gel works by correcting the acidic microenvironment and reducing inflammation in CEPC and NPC. It achieves this by inhibiting the expression of hypoxia‐inducible factor‐2 alpha (HIF‐2*α*) and transferrin receptor 1 (TfR1), while also decreasing the levels of intracellular ferric iron ions and ROS. Targeting inflammation in CEPC and NPC presents a promising therapeutic approach for IVDD [81].

## 12. HIF‐2*α* as a Therapeutic Target in Other Diseases

Hypoxia‐induced expression of HIF‐2*α* facilitates tumor growth and proliferation, and over the past 15 years, chronic inflammation has been determined to promote multiple hallmark functions in cancer [82]. Therefore, in cancer research, targeted inhibitors of HIF‐2*α* have become increasingly valuable. Bruick, Gardner, MacMillan, and Tambar identify a high‐affinity binding, potent, and subtype‐selective inhibitor of HIF‐2*α* in cancer cells: tetrazolium tetrahydro pyrimidine (S, R), a potent inhibitor of HIF‐2*α*, which provides a novel, artificial pathway for targeting deregulated HIF activity and lays the groundwork for ongoing studies of in vivo antagonism of HIF‐2*α* in animal models of cancer [83]. It has been shown that in breast cancer, HIF‐2*α* causes breast cancer cells (BCs) to transform into breast cancer stem cells (BCSCs), which are more resistant to treatment. YQ‐0629 can bind to the PAS‐B domain of HIF‐2*α*, inhibit the self‐renewal of BCSC, overcome BCSC chemoresistance, and inhibit tumor growth in vitro and in vivo, making it a promising candidate for targeted breast cancer therapy [84]. The association of HIF‐2*α* with the development of renal cancer has become increasingly valuable, the majority of which is ccRCC. Wallace and Chen described potent and selective specific antagonists of HIF‐2*α* in ccRCC, PT 2385, and PT 2399, respectively, in the same year, and clinical trials are underway [85–87]. Additionally, *Toona sinensis* (TS), a traditional Chinese herb, along with its leaf extract (TSL‐1), may inactivate the oncogenic pathway by inhibiting HIF‐2*α* protein expression in ccRCC cell lines [88]. Three years later, in a study of dynamic *N* ^−6^‐methyladenosine (^m6^A) modifications in hepatocellular carcinoma (HCC), PT 2385 achieved inhibition of HCC progression by reconstructing the epigenetic mechanism of YTH structural domain family 2 (YTHDF 2) programming [89]. More recently, Belzutifan (MK‐6482, formerly known as PT 2977), a potent and selective small‐molecule inhibitor of HIF‐2*α*, was studied in humans for the first time. The results demonstrated that Belzutifan was well‐tolerated and showed preliminary antitumor activity in heavily pretreated ccRCC patients and received U.S. Food and Drug Administration (FDA) approval in 2022 as the first HIF‐2*α* inhibitor for the treatment of VHL‐related diseases. [7, 8, 90, 91]. In 2025, Casdatifan (AB521) became the newest small molecule variant inhibitor of HIF‐2*α*, which, like YQ‐0629, also binds to the PAS‐B structural domain of HIF‐2*α*, inhibits HIF‐2*α* and HIF‐2*α* downstream target genes, VEGF and EPO, in HepG2, renal carcinoma cells, endothelial cells, and M2‐type macrophages, and was inactive against HIF‐1*α* [92].

**Figure 7:**
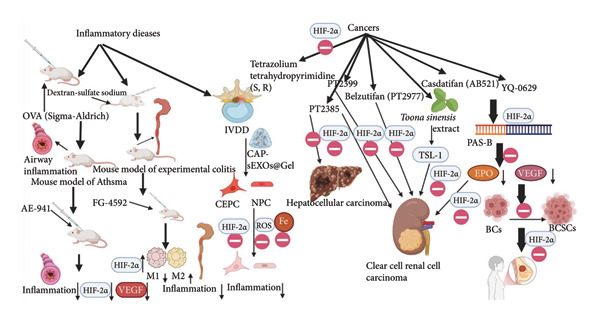
The use of HIF‐2*α* as a therapeutic target in a variety of diseases^13^ ^,^ ^14^.

Figure 7 summarizes the use of HIF‐2*α* as a therapeutic target in a variety of diseases. As illustrated in the figure, in inflammatory diseases, Neovastat (AE‐941) significantly reduced airway inflammation in asthmatic mice by inhibiting the expression of VEGF and HIF‐2*α*. Roxadustat improved symptoms in mice by increasing HIF‐2*α* levels, decreasing inflammatory cytokines, inhibiting M1 macrophage polarization, and enhancing M2 macrophage polarization in colonic tissues, thereby helping to treat colitis. The CAP‐sEXOs@Gel composite hydrogel reduces inflammation in cartilage endplate and NPC by regulating HIF‐2*α*/TfR 1, intracellular iron ions, and ROS. It offers an effective therapeutic option for IVDD. Tetrazolium tetrahydro pyrimidine (S, R) offers a novel artificial pathway that targets deregulated HIF activity, laying the groundwork for ongoing studies on in vivo antagonism of HIF‐2 in animal cancer models. PT 2385 can inhibit the progression of HCC. Additionally, PT 2385, PT 2399, TSL‐1, Belzutifan and Casdatifan are potent and selective small‐molecule inhibitors of HIF‐2*α*, which can effectively treat ccRCC. YQ‐0629 binds to the PAS‐B domain of HIF‐2*α*, preventing the transformation of BCs into BCSCs, thereby inhibiting tumor growth. It overcomes drug resistance associated with targeted therapies and holds promising therapeutic potential.

## 13. Conclusions

HIF‐2*α* is crucial in various inflammatory and hypoxic microenvironments. The factors determining its anti‐inflammatory or proinflammatory effects are complex. Most studies have shown that HIF‐2*α* mainly promotes M2 macrophage polarization in inflammatory cells, promotes or inhibits downstream inflammatory cytokine expression, and activates the NF‐κB signaling pathway. However, the specific mechanism of action of the HIF‐2*α* signaling pathway under inflammatory conditions and the key determinants affecting the anti‐inflammatory or proinflammatory effects of HIF‐2*α* are unknown. Therefore, the conclusive role of HIF‐2*α* in various inflammatory diseases cannot be defined at this moment. Additional research is needed to understand the available evidence, resolve any inconsistencies that arise, and confirm or deny the hypotheses presented. Notably, HIF‐2*α* has therapeutic potential, and targeted HIF‐2*α* inhibitors have shown promising therapeutic effects in both inflammation and cancer in animal models and even in humans. They should be applied in clinical trials as soon as possible to develop additional therapeutic strategies for more inflammatory diseases.

**Nomenclature**
APAP: Acetaminophen
Acss2: Acetate‐dependent acetyl coenzyme A synthase 2
AILI: Acute liver injury
ARDS: Acute respiratory distress syndrome
AJ: Adherens junction
AQP1: Aquaporin‐1
Arnt: Aromatic hydrocarbon receptor nuclear translocator
BC: Breast cancer cell
BCSC: Breast cancer stem cell
CEPC: Cartilage endplate cells
CK1δ: Casein kinase 1 Delta
ccRCC: Clear cell renal cell carcinoma
JNKs: c‐Jun NH 2‐terminal kinases
DMT1: Divalent metal ion transport protein 1
DCYTB: Duodenal cytochrome b561
^m6^A: Dynamic *N* ^−6^‐methyladenosine
EGL: Egg‐laying defective gene
EPO: Erythropoietin
ECM: Extracellular matrix
ERK: Extracellular signaling‐regulated kinase
FDA: Food and drug administration
HMLD: Hard metal lung disease
HCC: Hepatocellular carcinoma
HepG2: Hepatocellular carcinoma cell
HIF: Hypoxia‐inducible factor
HIF‐2α: Hypoxia‐inducible factor‐2*α*
HRE: Hypoxia response element
IBD: Inflammatory bowel disease
IVDD: Intervertebral disc degeneration
IL‐1β: Interleukin‐1β
IL‐6: Interleukin‐6
IL‐8: Interleukin‐8
FPN: Iron transport protein
JAK–STAT: Janus kinase–signal transducer and activator of transcription
LPS: Lipopolysaccharide
LIRI: Lung ischemia‐reperfusion injury
MIP: Macrophage inflammatory factor
MMP: Matrix metalloproteinase
mTOR: Mechanistic target of rapamycin
MIRI: Myocardial ischemia‐reperfusion injury
Plk1: Polo‐like kinase 1
PMN: Polymorphonuclear neutrophil
NLR: NOD (nucleotide‐binding oligomerization structural domain protein)‐like receptor
NASH: Nonalcoholic steatohepatitis
NF‐κB: Nuclear factor‐κB
NPC: Nucleus pulposus cells
OA: Osteoarthritis
PDLC: Periodontal ligament cell
PI3K: Phosphatidylinositol‐3 protein kinase
PHD: Prolyl‐4‐hydroxylase domain enzyme
Akt: Protein kinase B
PMVEC: Pulmonary microvascular endothelial cell
RHM: Recruited hepatic macrophage
ROS: Reactive oxygen species
GERD: Gastroesophageal reflux disease
TLR: Toll‐like receptor
TNF‐α: Tumor necrosis factor‐α
TAMs: Tumor‐associated macrophages
UUO: Unilateral ureteral obstruction
VEGF: Vascular endothelial growth factor
VE‐PTP: Vascular endothelial protein tyrosine phosphatase
VHL: Von Hippel–Lindau protein
YTHDF 2: YTH structural domain family 2

## Ethics Statement

The authors have nothing to report.

## Consent

The authors have nothing to report.

## Disclosure

All authors read and approved the final manuscript. Daohong Zhao is the corresponding author of this article.

## Conflicts of Interest

The authors declare no conflicts of interest.

## Author Contributions

All authors contributed equally to this work. Jiarui Huang was a major contributor in writing the manuscript.

## Funding

This review was supported by grants from Yunnan Revitalization Talent Support Program (XDYC‐QNRC‐2022‐0318), the Key Research and Development Program of Yunnan Provincial Science and Technology Department (202403AC100008), the First Class Discipline Team of Kunming Medical University (2024XKTDYS05), and Yunnan Province Spinal Malformation Prevention and Basic Research Innovation Team (202505AS350011).

## Endnotes

^1^This figure is an original figure created by Jiarui Huang.

^2^Created in BioRender. https://BioRender.com/k72v206.

^3^This figure is an original figure created by Jiarui Huang.

^4^Created in BioRender. https://BioRender.com/fv77x06.

^5^This figure is an original figure created by Jiarui Huang.

^6^Created in BioRender. https://BioRender.com/cz6lgb3.

^7^This figure is an original figure created by Jiarui Huang.

^8^Created in BioRender. https://BioRender.com/unuq40a.

^9^This figure is an original figure created by Jiarui Huang.

^10^Created in BioRender. https://BioRender.com/4nddt6e.

^11^This figure is an original figure created by Jiarui Huang.

^12^Created in BioRender. https://BioRender.com/maf3iqb.

^13^This figure is an original figure created by Jiarui Huang.

^14^Created in BioRender. https://BioRender.com/omxiifz.

## Data Availability

The data supporting this review article are from previously reported studies and datasets, which have been cited.

## References

[1] Malkov M. I. , Lee C. T. , and Taylor C. T. , Regulation of the Hypoxia-Inducible Factor (HIF) by Pro-Inflammatory Cytokines, Cells. (2021) 10, no. 9, 10.3390/cells10092340. PMC8466990 34571989

[2] Rosell-Garcia T. , Rivas-Munoz S. , Kin K. et al., Multimerization of HIF Enhances Transcription of Target Genes Containing the Hypoxia Ancillary Sequence, Biochim Biophys Acta Gene Regul Mech. (2023) 1866, no. 4, 10.1016/j.bbagrm.2023.194963. 37499936

[3] Cummins E. P. , Keogh C. E. , Crean D. , and Taylor C. T. , The Role of HIF in Immunity and Inflammation, Molecular Aspects of Medicine. (2016) 47-48, 24–34, 10.1016/j.mam.2015.12.004, 2-s2.0-84958118013. 26768963

[4] Mendoza S. V. , Genetos D. C. , and Yellowley C. E. , Hypoxia-Inducible Factor-2alpha Signaling in the Skeletal System, JBMR Plus. (2023) 7, no. 4, 10.1002/jbm4.10733. PMC10097641 37065626

[5] Liu H. , Xie Y. , Wang X. et al., Exploring Links Between 2-oxoglutarate-dependent Oxygenases and Alzheimer′s disease, Alzheimer′s & Dementia. (2022) 18, no. 12, 2637–2668, 10.1002/alz.12733. PMC10083964 35852137

[6] Strocchi S. , Reggiani F. , Gobbi G. , Ciarrocchi A. , and Sancisi V. , The Multifaceted Role of EGLN Family Prolyl Hydroxylases in Cancer: Going Beyond HIF Regulation, Oncogene. (2022) 41, no. 29, 3665–3679, 10.1038/s41388-022-02378-8. 35705735

[7] Cheng B. , Ma X. , Zhou Y. et al., Recent Progress in the Development of hypoxia-inducible Factor 2alpha (HIF-2alpha) Modulators: Inhibitors, Agonists, and Degraders (2009-2024), European Journal of Medicinal Chemistry. (2024) 275, 10.1016/j.ejmech.2024.116645. 38959730

[8] Ahmed R. and Ornstein M. C. , Targeting HIF-2 Alpha in Renal Cell Carcinoma, Current Treatment Options in Oncology. (2023) 24, no. 9, 1183–1198, 10.1007/s11864-023-01106-y. 37403008

[9] Brown E. , Rowan C. , Strowitzki M. J. et al., Mucosal Inflammation Downregulates PHD1 Expression Promoting a barrier-protective HIF-1alpha Response in Ulcerative Colitis Patients, The FASEB Journal. (2020) 34, no. 3, 3732–3742, 10.1096/fj.201902103R. 31944416

[10] Yu Z. , Ran G. , Chai J. , and Zhang E. E. , A nature-inspired HIF Stabilizer Derived from a highland-adaptation Insertion of Plateau Pika Epas1 Protein, Cell Reports. (2024) 43, no. 2024, 10.1016/j.celrep.2024.114727. 39269902

[11] Biswas S. , Troy H. , Leek R. et al., Effects of HIF-1alpha and HIF2alpha on Growth and Metabolism of Clear-Cell Renal Cell Carcinoma 786-0 Xenografts, Journal of Oncology. (2010) 2010, 10.1155/2010/757908, 2-s2.0-77955370669. PMC2905950 20652061

[12] Kapitsinou P. P. , Liu Q. , Unger T. L. et al., Hepatic HIF-2 Regulates Erythropoietic Responses to Hypoxia in Renal Anemia, Blood. (2010) 116, no. 16, 3039–3048, 10.1182/blood-2010-02-270322, 2-s2.0-77958177671. 20628150 PMC2974609

[13] Franke K. , Gassmann M. , and Wielockx B. , Erythrocytosis: the HIF Pathway in Control, Blood. (2013) 122, no. 7, 1122–1128, 10.1182/blood-2013-01-478065, 2-s2.0-84887130699. 23733342

[14] Mazumder S. , Higgins P. J. , and Samarakoon R. , Downstream Targets of VHL/HIF-alpha Signaling in Renal Clear Cell Carcinoma Progression: Mechanisms and Therapeutic Relevance, Cancers (Basel). (2023) 15, no. 4, 10.3390/cancers15041316. PMC9953937 36831657

[15] Chen J. , Chen J. , Fu H. et al., Hypoxia Exacerbates Nonalcoholic Fatty Liver Disease via the HIF-2alpha/PPARalpha Pathway, American Journal of Physiology. Endocrinology and Metabolism. (2019) 317, no. 4, E710–E722, 10.1152/ajpendo.00052.2019, 2-s2.0-85072994829. 31430204

[16] Mastrogiannaki M. , Matak P. , and Peyssonnaux C. , The Gut in Iron Homeostasis: Role of HIF-2 Under Normal and Pathological Conditions, Blood. (2013) 122, no. 6, 885–892, 10.1182/blood-2012-11-427765, 2-s2.0-84886902763. 23678007 PMC3743464

[17] Kaelin W. G.Jr, Cancer and Altered Metabolism: Potential Importance of hypoxia-inducible Factor and 2-oxoglutarate-dependent Dioxygenases, Cold Spring Harbor Symposia on Quantitative Biology. (2011) 76, no. 0, 335–345, 10.1101/sqb.2011.76.010975, 2-s2.0-84867420094. 22089927 PMC4197849

[18] Befani C. and Liakos P. , The Role of hypoxia-inducible factor-2 Alpha in Angiogenesis, Journal of Cellular Physiology. (2018) 233, no. 12, 9087–9098, 10.1002/jcp.26805, 2-s2.0-85050483500. 29968905

[19] Taylor C. T. and Scholz C. C. , The Effect of HIF on Metabolism and Immunity, Nature Reviews Nephrology. (2022) 18, no. 9, 573–587, 10.1038/s41581-022-00587-8. 35726016 PMC9208707

[20] Schwartz A. J. , Das N. K. , Ramakrishnan S. K. et al., Hepatic hepcidin/intestinal HIF-2alpha Axis Maintains Iron Absorption During Iron Deficiency and Overload, Journal of Clinical Investigation. (2019) 129, no. 1, 336–348, 10.1172/JCI122359, 2-s2.0-85059416200. 30352047 PMC6307944

[21] Ge H. , Zhu H. , Xu N. et al., Increased Lung Ischemia-Reperfusion Injury in Aquaporin 1-Null Mice Is Mediated via Decreased Hypoxia-Inducible Factor 2alpha Stability, American Journal of Respiratory Cell and Molecular Biology. (2016) 54, no. 6, 882–891, 10.1165/rcmb.2014-0363OC, 2-s2.0-84988822544. 26649797

[22] Ye C. , Qi W. , Dai S. et al., microRNA-223 Promotes Autophagy to Aggravate Lung ischemia-reperfusion Injury by Inhibiting the Expression of Transcription Factor HIF2alpha, American Journal of Physiology-Lung Cellular and Molecular Physiology. (2020) 319, no. 1, L1–L10, 10.1152/ajplung.00009.2020. 32267722

[23] Gong H. , Rehman J. , Tang H. et al., HIF2alpha Signaling Inhibits Adherens Junctional Disruption in Acute Lung Injury, Journal of Clinical Investigation. (2015) 125, no. 2, 652–664, 10.1172/JCI77701, 2-s2.0-84944180420. 25574837 PMC4319409

[24] Proper S. P. , Saini Y. , Greenwood K. K. et al., Loss of hypoxia-inducible Factor 2 Alpha in the Lung Alveolar Epithelium of Mice Leads to Enhanced Eosinophilic Inflammation in cobalt-induced Lung Injury, Toxicological Sciences. (2014) 137, 447–457, 10.1093/toxsci/kft253, 2-s2.0-84893357262. 24218148 PMC3908723

[25] Ramakrishnan S. K. and Shah Y. M. , Role of Intestinal HIF-2alpha in Health and Disease, Annual Review of Physiology. (2016) 78, no. 1, 301–325, 10.1146/annurev-physiol-021115-105202, 2-s2.0-84958644571. PMC4809193 26667076

[26] Brown E. and Taylor C. T. , Hypoxia-Sensitive Pathways in Intestinal Inflammation, The Journal of Physiology. (2018) 596, no. 15, 2985–2989, 10.1113/JP274350, 2-s2.0-85050955375. 29114885 PMC6068205

[27] Kerber E. L. , Padberg C. , Koll N. , Schuetzhold V. , Fandrey J. , and Winning S. , The Importance of Hypoxia-Inducible Factors (HIF-1 and HIF-2) for the Pathophysiology of Inflammatory Bowel Disease, International Journal of Molecular Sciences. (2020) 21, no. 22, 10.3390/ijms21228551. PMC7697655 33202783

[28] Triner D. , Xue X. , Schwartz A. J. , Jung I. , Colacino J. A. , and Shah Y. M. , Epithelial Hypoxia-Inducible Factor 2alpha Facilitates the Progression of Colon Tumors Through Recruiting Neutrophils, Molecular and Cellular Biology. (2017) 37, no. 5, 10.1128/MCB.00481-16, 2-s2.0-85013883423. PMC5311236 27956697

[29] Lin Q. and Yun Z. , The Hypoxia-Inducible Factor Pathway in Adipocytes: the Role of HIF-2 in Adipose Inflammation and Hypertrophic Cardiomyopathy, Frontiers in Endocrinology. (2015) 6, 10.3389/fendo.2015.00039, 2-s2.0-84926628423. PMC4369725 25852648

[30] Lin Q. , Huang Y. , Booth C. J. et al., Activation of hypoxia-inducible factor-2 in Adipocytes Results in Pathological Cardiac Hypertrophy, Journal of the American Heart Association. (2013) 2, no. 6, 10.1161/JAHA.113.000548, 2-s2.0-84902300494. PMC3886757 24326162

[31] Giaccia A. J. , HIF-2: the Missing Link Between Obesity and Cardiomyopathy, Journal of the American Heart Association. (2013) 2, no. 6, 10.1161/JAHA.113.000710, 2-s2.0-84902302570. PMC3886772 24342999

[32] Zhang F. J. , Luo W. , and Lei G. H. , Role of HIF-1alpha and HIF-2alpha in Osteoarthritis, Joint Bone Spine. (2015) 82, no. 3, 144–147, 10.1016/j.jbspin.2014.10.003, 2-s2.0-84929172313. 25553838

[33] Inoue H. , Arai Y. , Kishida T. et al., Hydrostatic Pressure Influences HIF-2 Alpha Expression in Chondrocytes, International Journal of Molecular Sciences. (2015) 16, no. 1, 1043–1050, 10.3390/ijms16011043, 2-s2.0-84964694467. 25569085 PMC4307289

[34] Souza R. F. , Bayeh L. , Spechler S. J. , Tambar U. K. , and Bruick R. K. , A New Paradigm for GERD Pathogenesis. Not Acid Injury, but cytokine-mediated Inflammation Driven by HIF-2alpha: a Potential Role for Targeting HIF-2alpha to Prevent and Treat Reflux Esophagitis, Current Opinion in Pharmacology. (2017) 37, 93–99, 10.1016/j.coph.2017.10.004, 2-s2.0-85032816842. 29112883 PMC5922421

[35] Herrero-Cervera A. , Soehnlein O. , and Kenne E. , Neutrophils in Chronic Inflammatory Diseases, Cell Molecular Immunology. (2022) 19, no. 2, 177–191, 10.1038/s41423-021-00832-3. PMC8803838 35039631

[36] Thompson A. A. , Elks P. M. , Marriott H. M. et al., Hypoxia-Inducible Factor 2alpha Regulates Key Neutrophil Functions in Humans, Mice, and Zebrafish, Blood. (2014) 123, no. 3, 366–376, 10.1182/blood-2013-05-500207, 2-s2.0-84893091765. 24196071 PMC3894493

[37] Murdoch C. , Muthana M. , and Lewis C. E. , Hypoxia Regulates Macrophage Functions in Inflammation, Journal of Immunology. (2005) 175, no. 10, 6257–6263, 10.4049/jimmunol.175.10.6257, 2-s2.0-27744603002. 16272275

[38] Sica A. and Mantovani A. , Macrophage Plasticity and Polarization: in Vivo Veritas, Journal of Clinical Investigation. (2012) 122, no. 3, 787–795, 10.1172/JCI59643, 2-s2.0-84857883847. 22378047 PMC3287223

[39] <Hypoxia-inducible factors as essential regulators of inflammation.pdf>. 10.1007/82_2010_74 PMC3144567 20517715

[40] Takeda N. , O′Dea E. L. , Doedens A. et al., Differential Activation and Antagonistic Function of HIF-Alpha Isoforms in Macrophages are Essential for NO Homeostasis, Genes & Development. (2010) 24, no. 5, 491–501, 10.1101/gad.1881410, 2-s2.0-77649177217. 20194441 PMC2827844

[41] Zhong B. , Du J. , Liu F. et al., Hypoxia-Induced factor-1alpha Induces NLRP3 Expression by M1 Macrophages in Noneosinophilic Chronic Rhinosinusitis with Nasal Polyps, Allergy. (2021) 76, no. 2, 582–586, 10.1111/all.14571. 32854144

[42] Imtiyaz H. Z. , Williams E. P. , Hickey M. M. et al., Hypoxia-Inducible Factor 2alpha Regulates Macrophage Function in Mouse Models of Acute and Tumor Inflammation, Journal of Clinical Investigation. (2010) 120, no. 8, 2699–2714, 10.1172/JCI39506, 2-s2.0-77955295091. 20644254 PMC2912179

[43] Li X. , Zhang X. , Xia J. et al., Macrophage HIF-2alpha Suppresses NLRP3 Inflammasome Activation and Alleviates Insulin Resistance, Cell Reports. (2021) 36, no. 2021, 10.1016/j.celrep.2021.109607. 34433035

[44] Jeelani I. , Moon J. S. , da Cunha F. F. et al., HIF-2alpha Drives Hepatic Kupffer Cell Death and Proinflammatory Recruited Macrophage Activation in Nonalcoholic Steatohepatitis, Science Translational Medicine. (2024) 16, no. 764, 10.1126/scitranslmed.adi0284. PMC11665927 39259813

[45] Choe S. S. , Shin K. C. , Ka S. , Lee Y. K. , Chun J. S. , and Kim J. B. , Macrophage HIF-2alpha Ameliorates Adipose Tissue Inflammation and Insulin Resistance in Obesity, Diabetes. (2014) 63, no. 10, 3359–3371, 10.2337/db13-1965, 2-s2.0-84907481906. 24947359

[46] Sen R. and Baltimore D. , Multiple Nuclear Factors Interact with the Immunoglobulin Enhancer Sequences, Cell. (1986) 46, no. 5, 705–716, 10.1016/0092-8674(86)90346-6, 2-s2.0-0022481133. 3091258

[47] <Prolyl hydroxylase-1 negatively regulates IkappaB kinase-beta, giving insight into hypoxia-induced NFkappaB activity.pdf>. 10.1073/pnas.0602235103 PMC1643842 17114296

[48] Oliver K. M. , Garvey J. F. , Ng C. T. et al., Hypoxia Activates NF-kappaB-dependent Gene Expression Through the Canonical Signaling Pathway, Antioxidants and Redox Signaling. (2009) 11, no. 9, 2057–2064, 10.1089/ars.2008.2400, 2-s2.0-68949207361. 19422287

[49] Lawrence T. , The Nuclear Factor NF-kappaB Pathway in Inflammation, Cold Spring Harbor Perspectives in Biology. (2009) 1, no. 6, 10.1101/cshperspect.a001651, 2-s2.0-77955906264. PMC2882124 20457564

[50] Gerondakis S. , Fulford T. S. , Messina N. L. , and Grumont R. J. , NF-kappaB Control of T Cell Development, Nature Immunology. (2014) 15, no. 1, 15–25, 10.1038/ni.2785, 2-s2.0-84890956580. 24352326

[51] Wullaert A. , Bonnet M. C. , and Pasparakis M. , NF-kappaB in the Regulation of Epithelial Homeostasis and Inflammation, Cell Research. (2011) 21, no. 1, 146–158, 10.1038/cr.2010.175, 2-s2.0-78650866071. 21151201 PMC3193399

[52] D′Ignazio L. , Batie M. , and Rocha S. , Hypoxia and Inflammation in Cancer, Focus on HIF and NF-kappaB, Biomedicines. (2017) 5, no. 2, 10.3390/biomedicines5020021, 2-s2.0-85029882313. PMC5489807 28536364

[53] Saito T. , Fukai A. , Mabuchi A. et al., Transcriptional Regulation of Endochondral Ossification by HIF-2alpha During Skeletal Growth and Osteoarthritis Development, Nature Medicine. (2010) 16, no. 6, 678–686, 10.1038/nm.2146, 2-s2.0-77953208836. 20495570

[54] Kobayashi H. , Gilbert V. , Liu Q. et al., Myeloid cell-derived hypoxia-inducible Factor Attenuates Inflammation in Unilateral Ureteral obstruction-induced Kidney Injury, Journal of Immunology. (2012) 188, no. 10, 5106–5115, 10.4049/jimmunol.1103377, 2-s2.0-84861128230. PMC3345098 22490864

[55] Kapitsinou P. P. , Sano H. , Michael M. et al., Endothelial HIF-2 Mediates Protection and Recovery from Ischemic Kidney Injury, Journal of Clinical Investigation. (2014) 124, no. 6, 2396–2409, 10.1172/JCI69073, 2-s2.0-84902172100. 24789906 PMC4092875

[56] He K. , Chen X. , Han C. et al., Lipopolysaccharide-Induced cross-tolerance Against Renal ischemia-reperfusion Injury is Mediated by hypoxia-inducible factor-2alpha-regulated Nitric Oxide Production, Kidney International. (2014) 85, no. 2, 276–288, 10.1038/ki.2013.342, 2-s2.0-84893322248. 24025643

[57] Willam C. , HIF Meets NF-kappaB Signaling, Kidney International. (2014) 85, no. 2, 232–234, 10.1038/ki.2013.362, 2-s2.0-84893232289. 24487361

[58] Cai H. , Bai Z. , and Ge R. L. , Hypoxia-Inducible factor-2 Promotes Liver Fibrosis in Non-alcoholic Steatohepatitis Liver Disease via the NF-kappaB Signalling Pathway, Biochemical and Biophysical Research Communications. (2021) 540, 67–74, 10.1016/j.bbrc.2021.01.002. 33450482

[59] Li W. , Wu N. , Wang J. , Wang Y. , Wu M. , and Wang H. , Role of HIF-2alpha/NF-kappaB Pathway in Mechanical stress-induced Temporomandibular Joint Osteoarthritis, Oral Diseases. (2022) 28, no. 8, 2239–2247, 10.1111/odi.13986. 34342085

[60] Gao R. Y. , Wang M. , Liu Q. et al., Hypoxia-Inducible Factor-2alpha Reprograms Liver Macrophages to Protect Against Acute Liver Injury Through the Production of Interleukin-6, Hepatology. (2020) 71, no. 6, 2105–2117, 10.1002/hep.30954. 31529728 PMC7075728

[61] Bae W. J. , Shin M. R. , Kang S. K. et al., HIF-2 Inhibition Supresses Inflammatory Responses and Osteoclastic Differentiation in Human Periodontal Ligament Cells, Journal of Cellular Biochemistry. (2015) 116, no. 7, 1241–1255, 10.1002/jcb.25078, 2-s2.0-84929119891. 25565665

[62] Yang Q. , Zhou Y. , Cai P. et al., Up-regulated HIF-2alpha Contributes to the Osteoarthritis Development Through Mediating the Primary Cilia Loss, International Immunopharmacology. (2019) 75, no. 2019, 10.1016/j.intimp.2019.105762, 2-s2.0-85069715597. 31357086

[63] Hu J. , Li X. , Yang L. , and Li H. , Hypoxia, a Key Factor in the Immune Microenvironment, Biomedicine & Pharmacotherapy. (2022) 151, 10.1016/j.biopha.2022.113068. 35676780

[64] Mantovani A. and Sica A. , Macrophages, Innate Immunity and Cancer: Balance, Tolerance, and Diversity, Current Opinion in Immunology. (2010) 22, no. 2, 231–237, 10.1016/j.coi.2010.01.009, 2-s2.0-77950944395. 20144856

[65] Joshi S. , Singh A. R. , Zulcic M. , and Durden D. L. , A macrophage-dominant PI3K Isoform Controls hypoxia-induced HIF1alpha and HIF2alpha Stability and Tumor Growth, Angiogenesis, and Metastasis, Molecular Cancer Research. (2014) 12, no. 10, 1520–1531, 10.1158/1541-7786.MCR-13-0682, 2-s2.0-84907964717. 25103499

[66] Mohlin S. , Hamidian A. , von Stedingk K. et al., PI3K-mTORC2 but Not PI3K-mTORC1 Regulates Transcription of HIF2A/EPAS1 and Vascularization in Neuroblastoma, Cancer Research. (2015) 75, no. 21, 4617–4628, 10.1158/0008-5472.CAN-15-0708, 2-s2.0-84946593823. 26432405

[67] Pangou E. , Befani C. , Mylonis I. et al., HIF-2alpha Phosphorylation by CK1delta Promotes Erythropoietin Secretion in Liver Cancer Cells Under Hypoxia, Journal of Cell Science. (2016) 129, no. 22, 4213–4226, 10.1242/jcs.191395, 2-s2.0-84995947052. 27686097

[68] Xu M. , Nagati J. S. , Xie J. et al., An Acetate Switch Regulates Stress Erythropoiesis, Nature Medicine. (2014) 20, no. 9, 1018–1026, 10.1038/nm.3587, 2-s2.0-84908323382. PMC4159437 25108527

[69] Chen R. , Xu M. , Nagati J. S. et al., The acetate/ACSS2 Switch Regulates HIF-2 Stress Signaling in the Tumor Cell Microenvironment, PLoS One. (2015) 10, no. 2, 10.1371/journal.pone.0116515, 2-s2.0-84923329578. PMC4331492 25689462

[70] Garcia J. A. , Chen R. , Xu M. et al., Acss2/HIF-2 Signaling Facilitates Colon Cancer Growth and Metastasis, PLoS One. (2023) 18, no. 3, 10.1371/journal.pone.0282223. PMC9980813 36862715

[71] Motzer R. J. , Jonasch E. , Agarwal N. et al., Kidney Cancer, Version 2.2017, NCCN Clinical Practice Guidelines in Oncology, Journal of the National Comprehensive Cancer Network. (2017) 15, no. 6, 804–834, 10.6004/jnccn.2017.0100, 2-s2.0-85020412401. 28596261

[72] Dufies M. , Verbiest A. , Cooley L. S. et al., Plk1, Upregulated by HIF-2, Mediates Metastasis and Drug Resistance of Clear Cell Renal Cell Carcinoma, Communications Biology. (2021) 4, no. 1, 10.1038/s42003-021-01653-w. PMC7865059 33547392

[73] Fedotcheva T. A. , Fedotcheva N. I. , and Shimanovsky N. L. , Progesterone as an Anti-inflammatory Drug and Immunomodulator: New Aspects in Hormonal Regulation of the Inflammation, Biomolecules. (2022) 12, no. 9, 10.3390/biom12091299. PMC9496164 36139138

[74] Nishimoto-Kakiuchi A. , Sato I. , Nakano K. et al., A long-acting anti-IL-8 Antibody Improves Inflammation and Fibrosis in Endometriosis, Science Translational Medicine. (2023) 15, no. 684, 10.1126/scitranslmed.abq5858. 36812343

[75] Fragoulis G. E. , Soulaidopoulos S. , Sfikakis P. P. , Dimitroulas T. , and G D. K. , Effect of Biologics on Cardiovascular Inflammation: Mechanistic Insights and Risk Reduction, Journal of Inflammation Research. (2021) 14, 1915–1931, 10.2147/JIR.S282691. 34017189 PMC8131071

[76] Garin N. , Sole N. , Lucas B. et al., Drug Related Problems in Clinical Practice: a cross-sectional Study on Their Prevalence, Risk Factors and Associated Pharmaceutical Interventions, Scientific Reports. (2021) 11, no. 1, 10.1038/s41598-020-80560-2. PMC7807048 33441854

[77] Stumpf M. A. M. , Pinheiro F. M. M. , Silva G. O. et al., How to Manage Intolerance to Dopamine Agonist in Patients with Prolactinoma, Pituitary. (2023) 26, no. 2, 187–196, 10.1007/s11102-023-01313-8. 37027090

[78] Lee S. Y. and Chung S. M. , Neovastat (AE-941) Inhibits the Airway Inflammation via VEGF and HIF-2 Alpha Suppression, Vascular Pharmacology. (2007) 47, no. 5-6, 313–318, 10.1016/j.vph.2007.08.009, 2-s2.0-35548940240. 17931982

[79] Kong G. , Hua H. , Lu Y. et al., Roxadustat Ameliorates Experimental Colitis in Mice by Regulating Macrophage Polarization Through Increasing HIF Level, Biochimica et Biophysica Acta (BBA)-General Subjects. (2024) 1868, no. 3, 10.1016/j.bbagen.2023.130548. 38158022

[80] Zhu H. , Zheng J. , Oh X. Y. et al., Nanoarchitecture-Integrated Hydrogel Systems Toward Therapeutic Applications, ACS Nano. (2023) 17, no. 9, 7953–7978, 10.1021/acsnano.2c12448. 37071059

[81] Zhan J. , Cui Y. , Zhang P. et al., Cartilage Endplate-Targeted Engineered Exosome Releasing and Acid Neutralizing Hydrogel Reverses Intervertebral Disc Degeneration, Advanced Healthcare Materials. (2025) 14, no. 2, 10.1002/adhm.202403315. 39555665

[82] Hanahan D. and Weinberg R. A. , Hallmarks of Cancer: the next Generation, Cell. (2011) 144, no. 5, 646–674, 10.1016/j.cell.2011.02.013, 2-s2.0-79952284127. 21376230

[83] Scheuermann T. H. , Stroud D. , Sleet C. E. et al., Isoform-Selective and Stereoselective Inhibition of Hypoxia Inducible Factor-2, Journal of Medicinal Chemistry. (2015) 58, no. 15, 5930–5941, 10.1021/acs.jmedchem.5b00529, 2-s2.0-84939192271. 26226049

[84] Yan Y. , He M. , Zhao L. et al., A Novel HIF-2alpha Targeted Inhibitor Suppresses hypoxia-induced Breast Cancer Stemness via SOD2-mtROS-PDI/GPR78-UPR(ER) Axis, Cell Death & Differentiation. (2022) 29, no. 9, 1769–1789, 10.1038/s41418-022-00963-8. 35301432 PMC9433403

[85] Wallace E. M. , Rizzi J. P. , Han G. et al., A Small-Molecule Antagonist of HIF2alpha is Efficacious in Preclinical Models of Renal Cell Carcinoma, Cancer Research. (2016) 76, no. 18, 5491–5500, 10.1158/0008-5472.CAN-16-0473, 2-s2.0-84988983212. 27635045

[86] Chen W. , Hill H. , Christie A. et al., Targeting Renal Cell Carcinoma with a HIF-2 Antagonist, Nature. (2016) 539, no. 7627, 112–117, 10.1038/nature19796, 2-s2.0-84994669316. 27595394 PMC5340502

[87] Cho H. , Du X. , Rizzi J. P. et al., On-target Efficacy of a HIF-2alpha Antagonist in Preclinical Kidney Cancer Models, Nature. (2016) 539, no. 7627, 107–111, 10.1038/nature19795, 2-s2.0-84994626953. 27595393 PMC5499381

[88] Chen Y. C. , Chien L. H. , Huang B. M. , Chia Y. C. , and Chiu H. F. , Aqueous Extracts of Toona sinensis Leaves Inhibit Renal Carcinoma Cell Growth and Migration Through JAK2/stat3, Akt, MEK/ERK, and mTOR/HIF-2alpha Pathways, Nutrition and Cancer. (2016) 68, no. 4, 654–666, 10.1080/01635581.2016.1158292, 2-s2.0-84966632734. 27115866

[89] Hou J. , Zhang H. , Liu J. et al., YTHDF2 Reduction Fuels Inflammation and Vascular Abnormalization in Hepatocellular Carcinoma, Molecular Cancer. (2019) 18, no. 1, 10.1186/s12943-019-1082-3. PMC6859620 31735169

[90] Choueiri T. K. , Bauer T. M. , Papadopoulos K. P. et al., Inhibition of hypoxia-inducible factor-2alpha in Renal Cell Carcinoma with Belzutifan: a Phase 1 Trial and Biomarker Analysis, Nature Medicine. (2021) 27, no. 5, 802–805, 10.1038/s41591-021-01324-7. PMC9128828 33888901

[91] Fallah J. , Brave M. H. , Weinstock C. et al., FDA Approval Summary: Belzutifan for Von Hippel-Lindau Disease-Associated Tumors, Clinical Cancer Research. (2022) 28, no. 22, 4843–4848, 10.1158/1078-0432.CCR-22-1054. 35727604 PMC9669093

[92] Schweickert P. G. , Piovesan D. , Mitchell C. G. et al., Casdatifan (AB521) Is a Novel and Potent Allosteric Small Molecule Inhibitor of Protumourigenic HIF-2alpha Dependent Transcription, British Journal of Pharmacology. (2025) 182, no. 17, 4147–4167, 10.1111/bph.70075. 40400177

